# Energy Efficient Real-Time Scheduling Using DPM on Mobile Sensors with a Uniform Multi-Cores

**DOI:** 10.3390/s17122906

**Published:** 2017-12-14

**Authors:** Youngmin Kim, Ki-Seong Lee, Chan-Gun Lee

**Affiliations:** 1 Da Vind College of Genneral Education, Chung-Ang University, Heuksuk-ro 84, Dongjak-gu, Seoul 06974, Korea; e010120302@cau.ac.kr (Y.K.); goory@cau.ac.kr (K.-S.L.); 2 Department of Computer Science and Engineering, Chung-Ang University, Heuksuk-ro 84, Dongjak-gu, Seoul 06974, Korea

**Keywords:** WSNs, mobile sensor, energy efficiency, DPM, T-Ler plane, real-time scheduling

## Abstract

In wireless sensor networks (WSNs), sensor nodes are deployed for collecting and analyzing data. These nodes use limited energy batteries for easy deployment and low cost. The use of limited energy batteries is closely related to the lifetime of the sensor nodes when using wireless sensor networks. Efficient-energy management is important to extending the lifetime of the sensor nodes. Most effort for improving power efficiency in tiny sensor nodes has focused mainly on reducing the power consumed during data transmission. However, recent emergence of sensor nodes equipped with multi-cores strongly requires attention to be given to the problem of reducing power consumption in multi-cores. In this paper, we propose an energy efficient scheduling method for sensor nodes supporting a uniform multi-cores. We extend the proposed T-Ler plane based scheduling for global optimal scheduling of a uniform multi-cores and multi-processors to enable power management using dynamic power management. In the proposed approach, processor selection for a scheduling and mapping method between the tasks and processors is proposed to efficiently utilize dynamic power management. Experiments show the effectiveness of the proposed approach compared to other existing methods.

## 1. Introduction

WSNs consist of a number of moblile sensor nodes which are tiny, multi-functional, and low-power. Table 1 lists mobile sensing platforms with various sensors. It is widely used in various applications to collect and process data, such as various types of physical and environment information. Recently, sensor nodes in WSNs have evolved for multimedia streaming and image processing. In response to these high performance demands, sensor nodes with multi-processors have emerged. A multi-processor sensor node platform, mPlatform, which is capable of parallel processing for computationally intensive signal processing, was proposed by Lymberopoulos et al. [1]. These platforms operate with limited batteries, as shown in Table 1. The use of a multi-cores in the sensor node makes energy consumption more serious. Power management among sensor nodes is of critical importance for several reasons: limited energy batteries and ensuring longevity [2,3,4], meeting performance requirements [2,5,6], inefficiency arising because of over provisioning resources [2], power challenges posed by CMOS scaling [2,7], and enabling green computing [2]. Recent advances in CMOS technology have improved the density and speeds for on-chip transistors. These trends limit the fraction of chips that can be used at maximum speeds within limited power. Therefore, power challenges in CMOS have been addressed for processor performance. Transistor performance scaling in the future may end if left unaddressed [8,9]. Battery-operated embedded systems are sensitive to high power consumption, which leads to heating and reduced battery lifetime. Thus, energy-efficient management is essential in several embedded systems such as wearable devices. Improving energy efficiency leads to scale performance without violating the power budget. In recent advances, mobile computing devices with a multi-cores have been dynamically increased for mobile convergence applications (e.g., video streaming and web browsing). Power management in embedded systems contributes to achieve nearly 3% of the overall carbon footprint in green computing [10]. Energy efficiency scheduling algorithms for the sensor node with multi-processors are necessary. The scheduling algorithms must be able to keep battery lifetime longer while meeting the time constraints.

The Asymmetric Multi-core Platform (AMP) is capable of parallelism with different performance levels. The examples of AMP include mobile phones, tablets, and high-end mobile sensor nodes. These devices are equipped with cores capable of handling tasks requiring high-performance processing. Note that not all tasks need the high-performance processing and energy efficient schemes are adopted even for the cores consuming low power. The problem of scheduling AMP for high-performance mobile sensors is important in terms of performance and energy efficiency. The scheduler can switch the high-performance cores to a low power state by assigning tasks to the low power cores when processing the tasks requiring low loads. It is also possible that powerful cores are changed into simpler cores to adapt the system to varying loads. ARM’s big.LITTLE architecture [17,18] is a representative example. In ARM’s big.LITTLE architecture, there are three modes for task migration: cluster migration, CPU migration, and global task scheduling. The scheduler improves energy efficiency by migrating tasks between big and little cores. In this paper, we discuss real-time scheduling problems in the context of AMP. We adopt a scheme using the T-Ler plane to develop energy-efficient scheduling algorithms for real-time tasks on uniform multi-core systems.

The T-Ler plane extends the T-L plane using a T-L abstraction strategy to fit uniform multi-core systems. The Voltage Frequency Scale (VFS) is exploited on energy-efficient scheduling algorithms using the T-L plane. On the other hand, there are not many studies related to Dynamic Power Management (DPM). Sensor network applications with varying loads depending on the situation can take advantage of the energy by switching the state of unnecessarily used processors. Kim et al. [19,20] proposed several T-L plane based energy-efficient algorithms using DPM for sensor nodes with identical multi-processors. However, these algorithms are not suitable for uniform multi-processor systems. In particular, it is hard to select the set of processors with the lowest power consumption among the multiple sets of processors that have the same capacity. We propose a new algorithm suitable for sensor node with uniform multi-processors, called Uniform-DPM. More specifically, we extend the previous approaches [19,20] by considering the characteristics of uniform multi-processors in terms of energy efficiency as follows:

- At the beginning of each T-Ler plane, select the processors operating with a low frequency and minimize the processing capacity as much as possible.
- Reduce the complexity of scheduling and fragments of idle time, and classify the processors and tasks into processor sets and task sets at the beginning of the T-Ler plane, respectively.
- At each event in the T-Ler plane, utilize constrained migration to reduce the complexity of scheduling and fragments of idle time.

The first extension is to reduce the power loss caused by uniform multi-processors that consist of processors with difference processing capacities. The previous approach [20], as shown in Section 2, focuses solely on minimizing the number of processors. It is not suitable for uniform multi-processors. In the case of uniform multi-processors, the processors must be selected considering the processing capacity and the frequency of each processor. The second extension is to classify processors and tasks for limited scheduling, where tasks in a set are only scheduled to processors in the according processor set. The third extension is to adjust the sets in each event and to assign tasks to the processors using the limited scheduling. These prevent the unnecessary migration of tasks and enables the collection of idle time on particular processors.

We organize this paper as follows. In Section 2, we introduce related works, including the approaches previously based on T-L plane targeting uniform multi-processors. In Section 3, we propose mechanisms to select processors and allocate tasks for energy-efficient scheduling in uniform multi-processors. We extend the proposed events in identical multi-processors to ones in uniform multi-processors. In Section 4, we perform experimental evaluations by comparing our proposed algorithms with other algorithms. Lastly, we present the conclusions and future works in Section 5.

## 2. Related works

### 2.1. Power Management Techniques

Due to the advancements in semiconductor process technologies, there have been more high-end processors available that integrate more transistors. Recently, real-time embedded systems have been increasingly adopting high-end processors. In addition, to improve the performance, real-time embedded systems are also adopting multi-processors. However, this increases the processor power consumption significantly. The power consumption of CMOS chips is as follows [21]:

(1) $$P_{total}=P_{static}+P_{dynamic}.$$

$P_{static}$ is the static power consumption which is calculated as the sum of the leakage power and short current power. $P_{dynamic}$ is the dynamic power consumption by charging and discharging of the output capacitance for processing time. It is not easy to reduce the static power consumption which depends on various parameters in the semiconductor process. Therefore, we focus on reducing the dynamic power consumption. Dynamic power is defined as:

(2) $$P_{dynamic}=\alpha CV^{2}f,$$

where *f* is the frequency, α is the switching activity factor, *V* is the supply voltage, and *C* is the capacitive load. DVFS is a method used to adjust the supply voltage and frequency of a CMOS chip by utilizing the slack time that occurs when scheduling tasks. On the other hand, DPM is a method of reducing energy consumption by switching to a low power state when slack time occurs. However, if a sufficient slack time is not guaranteed over the break-even time, the energy overhead caused by the state transition will cause loss. The break-even time $BET_{sleep}$ is determined by Equation (3) [22].

(3) $$BET_{sleep}=\max\left(t_{sw},\frac{E_{sw}-P_{sleep}\cdot t_{sw}}{P_{idle}-P_{sleep}}\right)$$

The transition energy overhead and recovery time are denoted as $E_{sw}$ and $t_{sw}$, respectively. $P_{idle}$ denotes the idle power. The sleep power is denoted by $P_{sleep}$. The break-even time should be considered when developing a scheduling algorithm that not only uses the sleep mode, but also guarantees real-time responsiveness.

### 2.2. Global Scheduling Approaches on Multi-Processors

Scheduling disciplines can be categorized by considering the complexity of the priority mechanisms and the degree of job migration. Considering how task priorities are determined, Carpenter et al. [23] have categorized the schemes to static, dynamic but fixed within a job, or fully dynamic.

- Static: A single fixed priority is applied to all jobs for each task in the system. e.g., Rate Monotonic (RM) scheduling.
- Dynamic but fixed within a job: Different priorities may be assigned for the jobs of a task, but a job has a fixed priority at different times. e.g., Earliest Deadline First (EDF) scheduling.
- Fully dynamic: Different priorities may be assigned for a single job at different times, e.g., Least Laxity First (LLF) scheduling.

Depending on the degree of job migration, Carpenter et al. [23] have categorized the migration criterion to no migration, restricted migration, and unrestricted migration.

- No migration: The set of tasks in the system is partitioned into some subsets for available processors, a scheduler schedules a subset on a unique processor. The jobs of a task in a subset are executed on the corresponding processor.
- Restricted migration: Each job of a task must be scheduled entirely on a single processor. However, other jobs of the same task may be executed on different processors. Therefore, migrations among processors are allowed at the task-level context, but not at job boundaries.
- Unrestricted migration: Any jobs is also allowed to migrate among processors during its lifetime.

Note that our proposed scheduling algorithm supports fully dynamic and unrestricted migration.

**Figure 1:**
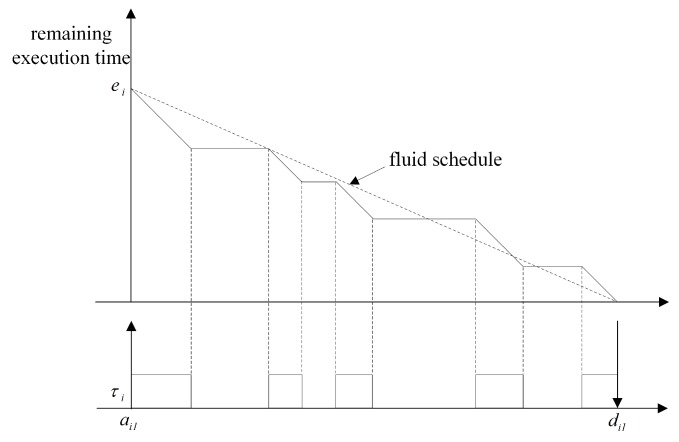
Fluid schedule model.

**Figure 2:**
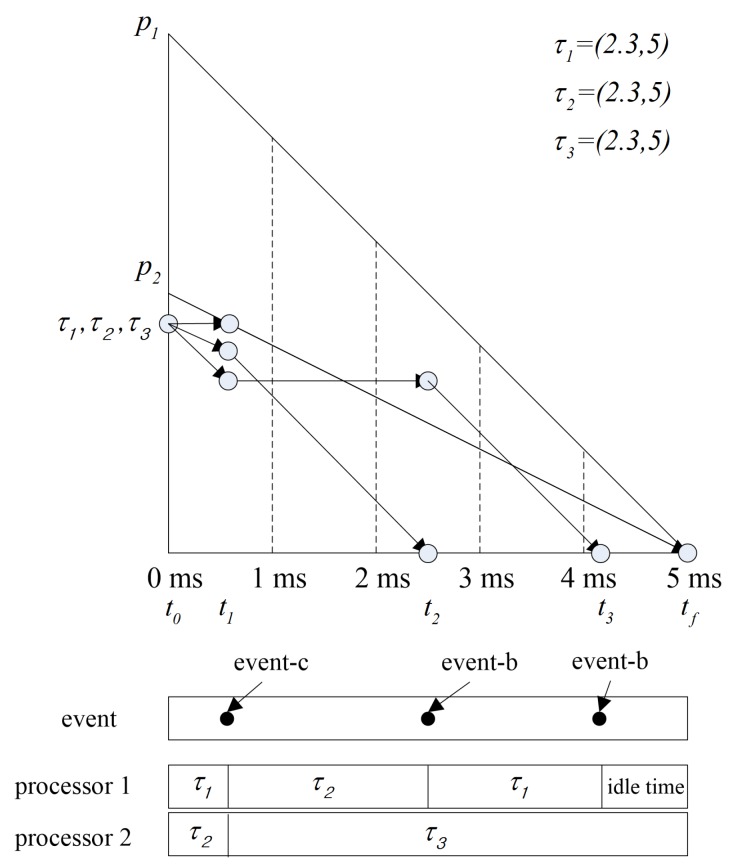
A scheduling example in the 1st T-Ler plane.

Various global scheduling algorithms for multi-processors have been studied. In global scheduling, all eligible jobs waiting for execution are in a single priority-ordered queue shared by all of the processors in the system; the highest priority job is dispatched from this queue by the global scheduler. Most of early the studies on global scheduling extended optimal scheduling algorithms known well for a single processor, such as RM and EDF, to multi-processors. However, these extensions can result in wasted utilization of resources. The fluid scheduling model with fairness notion, where each task is always executed at a fixed rate, emerged to overcome the limitation [24]. Figure 1 compares the fluid scheduling concept and the practical scheduling. There is a gap between fluid scheduling and practical scheduling, as shown in Figure 1. There are some algorithms extending the fluid scheduling model for achieving optimality on multi-processors. Proportionate fair (P-fair) scheduling has produced a feasible schedule for periodic tasks on multi-processors, and it has shown considerable promise in multi-processor scheduling [25]. However, extensive amount of migrations and preemption are needed to follow the fluid schedule. Much effort has been made to overcome this problem in global optimal scheduling. Thereafter, Deadline Partitioning-fair (DP-fair) and Deadline Partitioning-warp (DP-wrap) algorithms were proposed, and they exhibited better performance with respect to preemption in [26]. The method of allocating tasks to the processors supported by these scheduling algorithms is not suitable for uniform multi-processors. Cho et al. [27] proposed Largest Nodal Remaining Execution-time First (LNREF) using a T-L plane abstraction and it performs well with uniform multi-processors. Funk and Meka [28] proposed a T-L plane based scheduling algorithm, U-LLREF, that extends LNREF algorithm for uniform parallel machines. In U-LLREF, a uniform multi-processors provides a condition for determining event-c. Chen et al. [29] proposed Precaution Cut Greedy (PCG), a T-L plane based scheduling algorithm for uniform multi-processors. PCG uses a modified T-L plane, a T-Ler plane. Figure 2 shows how the PCG schedules in the first T-Ler plane. When event-c occurs, $\tau_{3}$ is assigned to $p_{2}$ until the end of the T-Ler plane. Thus, in PCG, a task monopolizes a single processor, thereby preventing unnecessary task migration.

### 2.3. T-L Plane Based Energy-Efficient Global Optimal Scheduling Approaches

**Figure 3:**
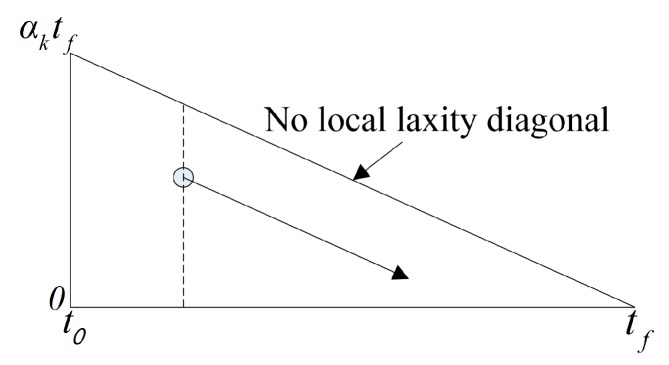
Transistion of the T-L plane (frequency $=\alpha_{k}$).

Energy-efficient scheduling based on the T-L plane for uniform parallel machines has been proposed due to the demand for energy efficiency. Uniform RT-SVFS [30] reduces the energy consumption by scaling the frequency of all processors with a constant rate. By scaling the height of the T-L plane, as shown in Figure 3, scheduling is enabled at the changed frequency. $\alpha_{k}$ represents the normalized frequency of the processor. In addition, energy-efficient T-L plane based scheduling algorithms for unrelated parallel machines have emerged. Independent RT-SVFS [30] determines the frequency by statically scaling each processor. This algorithm has been proposed to overcome the heavy task bottlenecks that can occur when using the frequency scaling technique. The Growing Minimum Frequency (GMF) [31], which is a state-of-the-art algorithm for T-L plane based non-uniform frequency scaling for saving energy on VFS embedded multi-processors, has been proposed for the frequency control of multi-processors using U-LLREF, and the global optimal frequency can be determined. RT-DVFS [32] allows you to dynamically adjust the frequency of each processor in the event of scheduling.

It is difficult to consider DPM due to the idle time fragmentation problem that occurs when using the T-L plane based scheduling algorithm. In addition, since scheduling is performed using all processors existing in the system, a considerable energy overhead due to unnecessary state transition occurs when DPM is used. TL-DPM [19] solves the idle time fragmentation problem by using a new event to retrieve tokens, which is performed in the next plane. However, since only the token of the next plane is targeted, there is room for solving the idle time fragmentation problem. Kim et al. [20] proposed a generalized method for executing tokens to be scheduled in the later plane in the current plane in order to solve this problem. To reduce the number of state transitions, scheduling is performed using only the minimum number of processors.

## 3. Proposed Energy Efficient Approach on Uniform Multi-Processors

### 3.1. Feasibility Conditions

Theorems 1 and 2 represent the conditions that must be met to obtain schedules satisfying the time constraints when uniform multi-processors are used for scheduling the given task set.

**Theorem** **1.** *(Horvath et al. [33]) The level algorithm constructs a minimal length schedule for the set of independent tasks with service requirements $e_{1}\geq e_{2}\geq \ldots \geq e_{n}$ on the processing system $\pi =(c_{1}\geq c_{2}\geq \ldots \geq c_{m})$, where m≤n. The schedule length is given by*

(4) $$\max\{\max_{1\leq i\leq m}\left(\frac{e_{i}}{c_{i}}\right),\frac{e_{n}}{c_{m}}\}.$$

**Theorem** **2.** *(Funk et al. [34]) Consider a set $\tau =\{\tau_{1},\ldots ,\tau_{n}\}$ of periodic tasks indexed according to non-increasing utilization (i.e., $u_{i}\geq u_{i+1}$ for all i, 1≤i≤n, where $u_{i}=e_{i}/p_{i}$). Let $U_{i}=\sum_{j=1}^{i}u_{i}$ for all i, 1≤i≤n. Let π denote a system of m≤n uniform processors with capacities $c_{1}$, $c_{2}$, …, $c_{m}$, $c_{i}\geq c_{i+1}$ for all i, 1≤i≤m. Periodic task system τ can be scheduled to meet all deadlines on the uniform multi-processor platform π if and only if the following constraints hold:*

(5) $$U_{n}\leq c_{m},$$

*where $U_{k}\geq c_{k}$, for all k=1,2,…,m.*

Selecting processors for the scheduling tasks at the beginning of each T-L plane is divided into the case where tasks are allocated to the processors with the same capacity as the utilization, and the case where they are not.

### 3.2. Processor Selections and Classification

#### 3.2.1. Simple Case: Exact Match

Table 2 shows some examples of processor selections for scheduling the task set shown in Table 3. In a CMOS chip, power consumption is determined by the operating frequency and supply voltage. The relationship between the power consumption and the supply voltage in the processor is as follows.

(6) $$P_{dynamic}\propto V^{2}.$$

In addition, according to the relationship between the supply voltage and the operating frequency in a processor, shown in Equation (7), a processor operating at a higher frequency requires a higher supply voltage than that operating at a lower frequency. Therefore, as shown in Table 2, a processor with a higher supply voltage will have a higher capacity.

(7) $$\;f\propto \frac{{\left(V-V_{th}\right)}^{\beta }}{V},$$

where $V_{th}$ is the threshold voltage of transistors and β is a measure of the velocity saturation in COMS transistors.

$S_{1}$, $S_{2}$, and $S_{3}$ shown in Table 2 satisfy Theorems 1 and 2 presented above. Since the processing capacity of $S_{1}$, $S_{2}$, and $S_{3}$ is equal to the total utilization of the task set, there is no idle time when the task set is scheduled. However, since the number and capacity of processors is not the same in each processor, the power consumed by $S_{1}$, $S_{2}$, and $S_{3}$ is different. The energy consumption for scheduling the task set in Table 3 on $S_{1}$, $S_{2}$, and $S_{3}$ is shown in Table 4. The lowest power consumption can be observed on $S_{2}$, where each task is independently assigned to a processor whose capacity is equal to the utilization of each task in the task set. If the total capacity of a processor set is equal to the total utilization of a task set, then there is no idle time because all processors always perform their tasks. Therefore, the power consumption of each processor is dependent on the processed workload. High-capacity processors can handle more work in terms of processor workloads. Equation (8) shows the power consumption $E_{e}\left(V_{k}\right)$ needed to process $e_{i}$ in a processor whose operating frequency and supply voltage are $f_{k}$ and $V_{k}$, respectively.

(8) $$\;E_{e}\left(V_{k}\right)=\alpha CV_{k}^{2}f_{k}\left(\frac{e_{i}}{f_{k}}\right)=\alpha CV_{k}^{2}e_{i},$$

where $V_{k}$ and $f_{k}$ denote the voltage and capacity of the *k-th* processor, respectively. Lemma 1 shows the power consumption characteristics of processor sets whose total capacity is equal to the total utilization of a task set.

**Lemma** **1.** If $U_{total}=c_{n}=c_{i}+c_{j}$, when scheduling a task set with $U_{total}$ on two processor sets $S_{1}=\left\{c_{n}\right\}$ and $S_{2}=\left\{c_{i},c_{j}\right\}$, the power consumption satisfies $\alpha CV_{n}^{2}e_{n}>\alpha CV_{i}^{2}e_{i}+\alpha CV_{j}^{2}e_{j}$.

**Proof of Lemma** **1.** According to Equation (8), the power consumption measures of $S_{1}$ and $S_{2}$ are $\alpha CV_{n}^{2}e_{n}$ and $\alpha CV_{n}^{2}e_{i}+\alpha CV_{i}^{2}e_{j}$ respectively. Since $c_{n}=U_{total}$ and $c_{i}+c_{j}=U_{total}$, there is no idle time when the tasks are scheduled. In addition, $V_{n}^{2}e_{n}=V_{n}^{2}\left(e_{i}+e_{j}\right)>V_{i}^{2}e_{i}+V_{j}^{2}e_{j}$, where $e_{n}=e_{i}+e_{j}$ and $V_{n}>V_{i},V_{j}$. Hence, $\alpha CV_{n}^{2}e_{n}>\alpha CV_{i}^{2}e_{i}+\alpha CV_{j}^{2}e_{j}$. ☐

According to Lemma 1, selecting the 0.8 capacity processor for scheduling the 0.6 and 0.2 utilization tasks in the task set, as shown in Table 1, will result in higher power consumption than selecting the 0.6 and 0.2 capacity processors for the scheduling the tasks. Therefore, assigning each task to the processor sets whose capacity is equal to its utilization is the most energy-efficient way when there are enough processors. Under the condition of $c_{i}\leq u_{i}$, the processor whose capacity is equal to $u_{i}$ shows the lowest power consumption to execute the task with the utilization $u_{i}$. Lemma 2 shows these characteristics.

**Lemma** **2.** When a task with utilization $u_{i}$ is executed on two processors under the condition of $c_{n}>c_{j}=u_{i}$, their power consumption for processing the allocated workload during the task period is $E_{e}\left(V_{n}\right)>E_{e}\left(V_{j}\right)$.

**Proof of Lemma** **2.** When two processors with capacities of $c_{n}$ and $c_{j}$ perform the workload $e_{i}$ during the period $p_{i}$, their power consumption measures are $E_{e}\left(V_{n}\right)$ and $E_{e}\left(V_{j}\right)$ respectively. If $c_{n}>c_{j}$, $V_{n}>V_{j}$ is satisfied by Equation (7). Hence, $E_{e}\left(V_{n}\right)>E_{e}\left(V_{j}\right)$ is satisfied by Equation (8). ☐

When a task set with the total time of $\sum_{i=1}^{n}e_{i}$ is scheduled on *n* processors whose capacity is different, the power consumption required for processing the allocated workload on n processors is shown in Equation (9). $e_{1}$, $e_{2}$, …, $e_{n}$ represents the workload assigned to each processor.

(9) $$\begin{array}{cc} E_{e} & =\alpha CV_{1}^{2}f_{1}\left(\frac{e_{1}}{f_{1}}\right)+\alpha CV_{2}^{2}f_{2}\left(\frac{e_{2}}{f_{2}}\right)+\ldots +\alpha CV_{n}^{2}f_{n}\left(\frac{e_{n}}{f_{n}}\right)\\ & =\alpha CV_{1}^{2}e_{1}+\alpha CV_{2}^{2}e_{2}+\ldots +\alpha CV_{n}^{2}e_{n}\\ & =\sum_{i=1}^{n}E_{e}\left(V_{i}\right). \end{array}$$

If the total capacity of n processors is greater than the total utilization of a task set to be scheduled, there will be idle time during task scheduling. This means that the power consumption during the idle time should be taken into account to measure the processors’ power consumption required for scheduling the task set. The power consumption of *n* processors is shown in Equation (10). $\alpha_{i}$ denotes the power consumption of the *i*-th processor during the idle time.

(10) $$\;E_{d}=\sum_{i=1}^{n}\left(E_{e}\left(V_{i}\right)+\alpha_{i}\right).$$

Lemma 3 and Theorem 3 show the power consumption required for scheduling a task set on a set of n processors with different capacities.

**Lemma** **3.** When the task set is scheduled with the processor set $S_{1}$, the lowest power is consumed, where the total capacity of $S_{1}$ is $\sum_{\forall \tau_{i}\in \tau }u_{i}=\sum_{\forall p_{i}\in S_{1}}c_{i}$.

**Proof of Lemma** **3.** If $\sum_{\forall \tau_{i}\in \tau }u_{i}=\sum_{\forall p_{i}\in S_{1}}c_{i}$, scheduling involves no idle time, so the processor power consumption is $\sum_{\forall p_{i}\in S_{1}}E_{e}\left(V_{i}\right)$. If $\sum_{\forall \tau_{i}\in \tau }u_{i}<\sum_{\forall p_{i}\in S_{1}}c_{i}$, scheduling involves some idle time, so the processor power consumption based on Equation (10) is $\sum_{\forall p_{i}\in S_{1}}\left(E_{e}\left(V_{i}\right)+\alpha_{i}\right)$. Hence, if $\sum_{\forall \tau_{i}\in \tau }u_{i}=\sum_{\forall p_{i}\in S_{1}}c_{i}$, then the lowest power consumption will be observed. ☐

**Theorem** **3.** Independently assigning each task in the task set τ to processors whose capacity is equal to the utilization of the task $u_{i}$ shows the lowest power consumption for scheduling a set of tasks.

**Proof of Theorem** **3.** This is easily proven by Lemmas 1–3. ☐

Therefore, selecting processors whose capacity is equal to the utilization of each task shows the lowest power consumption for scheduling a set of tasks.

#### 3.2.2. Generalized Solution

When not assignable to a processor with the same capacity as the task’s utilization, it is necessary to select a processor set available for scheduling with the limited processors. Table 5 shows the characteristics of processors used for task scheduling. Table 6 shows the processor sets selected from the processors in Table 5 for scheduling the task set shown in Table 7.

Since the processor sets $S_{1}$, $S_{2}$, $S_{3}$, and $S_{6}$ shown in Table 6 satisfy Theorems 1 and 2, they can be used for task scheduling. However, since the processor sets are differently configured, the idle time during the task scheduling and the difference in their supply voltages result in their different power consumption. Therefore, the following two strategies should be considered to select energy-efficient processors.

Selecting a processor set for task scheduling in consideration of all the problems presented above is a NP-hard problem. Therefore, in this paper, we propose a heuristic method for selecting an energy-efficient processor set. In the proposed method, if the size of the current plane is smaller than $C_{sleep}$, the processor in active mode is added to a processor set for task scheduling because it cannot be switched to sleep mode at the end of the previous plane. If the preferentially selected processors are not enough for scheduling the given task set, additional processors will be selected. Processors for scheduling are selected in terms of the local utilization of tasks from highest to lowest. Selecting processors for scheduling depends on the difference between the total local utilization of tasks in $\tau_{ready}$ at the start time $t_{0}$ in each plane $\sum_{\tau_{j}\in \tau_{ready}}r_{j}\left(t_{0}\right)$ and the total capacity $\sum_{p_{j}\in P_{selected}}c_{j}$ of the processors in $P_{selected}$. The selected processors are moved to $P_{selected}$. The following describes how to select processors.

- If $0\leq \sum_{\tau_{j}\in \tau_{ready}}r_{j}\left(t_{j}\right)-\sum_{p_{j}\in P_{selected}}c_{j}<r_{i}\left(t_{0}\right)$, the processor with the smallest capacity is selected for scheduling in the given processor set, $\left\{p_{j}|c_{j}\geq r_{i}\left(t_{0}\right)-\sum_{\tau_{j}\in \tau_{ready}}r_{j}\left(t_{0}\right)-\sum_{p_{j}\in P_{selected}}c_{j}\;where\;p_{j}\in \left\{P_{all}-P_{selected}\right\}\right\}$.
- If $\sum_{\tau_{j}\in \tau_{ready}}r_{j}\left(t_{0}\right)-\sum_{p_{j}\in P_{selected}}c_{j}\geq r_{i}\left(t_{0}\right)$, the previously selected processor is used for scheduling without selecting an additional processor.

$P_{all}$ is the set of all the processors in the system. $P_{selected}$ is the set of the selected processors for task scheduling. Algorithm 1 shows how to select processors for scheduling at the beginning of each plane. The function *getMinimumCapacityProcessor(availableCapacity, τ, $P_{temp}$)* takes the available capacity (*availableCapacity*) of the previously selected processor into account to return the lowest capacity processor for scheduling the task set τ from the given processor set $P_{temp}$. The function *add*() adds elements to the set, and the function *erase*() removes elements from the set. The processors in $P_{sleep}$ indicate the processor in the sleep state in the plane. It is necessary to ensure a break-even time longer than the idle time in order to use DPM techniques for switching the state of a processor. To ensure the idle timeis long enough to enter the sleep mode, the idle time in the plane should be generated as much as possible on a single processor. To prevent unnecessary power consumption, a task is assigned to the selected processor whose capacity is the lowest for scheduling the task. For this reason, in the proposed method, the processors in the selected processor set are classified into the following categories: processors that can be used to the maximum extent in the plane and processors that can be used exclusively by a single task in the plane. That is, the processors in $P_{selected}$ are classified into the following categories: $P_{fixed}$, $P_{max}$, and $P_{slack}$. The processors in $P_{fixed}$ represent a set of processors exclusively used by a single task. $P_{max}$ is the set of processors used to the maximum extent in the plane. $P_{slack}$ is the set of processors that may result in idle time in the plane during task scheduling. The tasks to be executed on the classified processor sets are divided into the following categories: $\tau_{fixed}$, $\tau_{max}$, and $\tau_{slack}$. Tasks assigned to a processor set cannot be moved to another processor set. The following describes how to classify the processor sets.

**Algorithm 1** Processor selection of the beginning time of a T-L plane  1:**Input**: $P_{all},P_{sleep},\tau_{all},psize$  2:**Output**: $P_{selected},\tau_{ready}$  3:psize—Size of the T-L plane  4:$P_{all}$—The set of processors in the system  5:$P_{sleep}$—The set of processors to be sleep mode  6:$P_{selected}$—The set of processors selected for scheduling tasks  7:$P_{temp}$—The temporary set of processors  8:$\tau_{all}$—The set of all tasks in the system  9:$\tau_{ready}$—The set of ready tasks10:τ—Temporary variable for tasks11:*p*—Temporary variable for processors12:*availableCpacity*—Temporary variable for available capacity13:**for** $\forall p\in P_{all}-P_{sleep}$ **do**14:    **if** $psize<p.C_{sleep}$ **then**15:        *add*($p,P_{temp}$);16:    **end if**17:**end for**18:**for** $\forall \tau \in \tau_{all}$ **do**19:    **if** τ.e>0 **then**20:        *add*($\tau ,\tau_{ready}$);21:    **end if**22:**end for**23:**repeat**24:    τ = *getFirstLocalUtilizationTask*($\tau_{ready}$);25:    *availableCapacity* = $\sum_{p_{i}\in P_{selected}}p_{i}.c-\sum_{\tau_{i}\in \tau_{ready}}\tau_{i}.r\left(t_{0}\right)$26:    **if** $\mathrm{availableCapacity}\geq \tau .r(t_{0})$ **then**27:        contitue;28:    **else**29:        *p* = *getMinimumCapacityProcessor*($availableCapacity,\tau ,P_{temp}$);30:        **if** *p* is null **then**31:           *p* = *getMinimumCapacityProcessor*($availableCapacity,\tau ,P_{sleep}$);32:           **if** $p.c>p_{1}.c$ **then**33:               *erase*($p,P_{sleep}$)34:           **end if**35:           *erase*($p,P_{sleep}$);36:        **else**37:           *erase*($p,P_{temp}$);38:        **end if**39:        *add*($p,P_{selected}$);40:        $p_{1}=p$;41:    **end if**42:**until** τ is not null43:**return** $P_{selected},\tau_{ready}$

1. To select a processor for scheduling a task $\tau_{i}$ in $\tau_{ready}$ where the difference between the total local utilization of the tasks in $\tau_{slack}$ at $t_{0}$ ($\sum_{\tau_{j}\in \tau_{slack}}\left(t_{0}\right)$) and the total capacity of the processors in $P_{slack}$ ($\sum_{\tau_{j}\in \tau_{slack}}r_{j}$) is greater than zero: $\sum_{\tau_{j}\in \tau_{slack}}r_{j}\left(t_{0}\right)-\sum_{p_{j}\in P_{slack}}c_{j}>0$.
  - If $\sum_{\tau_{j}\in \tau_{slack}}r_{j}\left(t_{0}\right)-\sum_{p_{j}\in P_{slack}}c_{j}\geq r_{i}\left(t_{0}\right)$, the task is additionally assigned to a previously selected processor without selecting an additional processor. The assigned task is moved from $\tau_{slack}$ to $\tau_{ready}$.
  - If $\sum_{\tau_{j}\in \tau_{slack}}r_{j}\left(t_{0}\right)-\sum_{p_{j}\in P_{slack}}c_{j}<r_{i}\left(t_{0}\right)$, the task is additionally assigned to a previously selected processor without selecting an additional processor. The assigned task is moved from $\tau_{slack}$ to $\tau_{ready}$.
2. If $\sum_{\tau_{j}\in \tau_{slack}}r_{j}\left(t_{0}\right)-\sum_{p_{j}\in P_{sl}}c_{j}=r_{i}\left(t_{0}\right)$,
  - All processors and tasks in $P_{slack}$ and $\tau_{slack}$ are moved to $P_{max}$ and $\tau_{max}$.
  - The processor whose capacity is the lowest for scheduling a task $\tau_{i}$, is selected from the following processor set, $\left\{p_{j}|c_{j}\geq r_{i}\left(t_{0}\right)wherep_{j}\in P_{selected}\right\}$. If the capacity of the selected processor is equal to the local utilization ($r_{i}\left(t_{0}\right)$) of the task $t_{i}$, the processor and the task are moved to $P_{fixed}$ and $\tau_{fixed}$, respectively. Otherwise, they are moved to $P_{slack}$ and $\tau_{slack}$, respectively.

Algorithm 2 shows how to classify the processor set $P_{selected}$ into the following categories: $P_{fixed}$, $P_{max}$, and $P_{slack}$. The task with the highest local utilization is considered first to classify the processor set and the task set. The function *getFirstLocalUtilizationTask*($\tau_{ready}$) returns the task with the highest local utilization in $\tau_{ready}$. The function *getMinimumCapacityProcessor*(*availableCapacity, τ, $P_{selected}$*) takes *availableCapacity* into account to return the processor whose capacity is the lowest for scheduling a task τ in $P_{selected}$. If the capacity of the returned processor is equal to the local utilization of the task, the processor and the task is moved to $P_{slack}$ and $\tau_{slack}$, respectively. If *availableCapacity* is 0, the processors in $P_{slack}$ and the tasks in $\tau_{slack}$ are moved to $P_{max}$ and $\tau_{max}$.

### 3.3. Scheduling Strategy

In the paper written by Chen et al. [29] , there are two suggested methods for scheduling on a uniform multi-processors. However, event-t, event-s, and event-r presented above are not taken into account in these scheduling methods. In this section, we propose a new T-L plane based scheduling method in which event-t, event-s, and event-r are used to reduce the power consumption of a uniform multi-processors. When the $\tau_{fixed}$, $\tau_{max}$, and $\tau_{slack}$ tasks are scheduled with the $P_{fixed}$, $P_{max}$, and $P_{slack}$ processor sets, the tasks cannot be moved from one processor set to another in order to generate no idle time on the processors in $P_{fixed}$ and $P_{max}$. The remaining part shows the movement of elements between task sets and processor sets and the processor assignment when a rescheduling event occurs. Since event-t as defined above targets identical multi-processors is not suitable for uniform multi-processors, it is redefined as in Definition 1.

**Definition** **1.** An event-t in uniform multi-processors occurs at $t_{t}$ if the following conditions are met.

- $t_{f}-t_{t}\geq C_{sleep}$.
- $\sum_{\tau_{i}\in \tau_{active}}r_{i}\left(t_{t}\right)=\left(\sum_{p_{i}\in P_{slack}}c_{i}\right)-c_{j}$ where $p_{j}\in P_{slack}$.

Algorithm 3 shows the process of assigning tasks to processors when a rescheduling event occurs. All the tasks in the set $\tau_{active}$ are moved to the set $\tau_{ready}$, and all the tasks in the set $\tau_{active}$ are deleted. The function *eraseAll*($\tau_{active}$) removes all elements in the set $\tau_{active}$. Tasks are assigned to processors in each processor set in the following order: $P_{slack}$, $P_{max}$, and $P_{fixed}$. The function *getMaximumLocalUtilizationTask*(*p.c*, $\tau_{fixed}$, $\tau_{ready}$) returns the task with the highest local utilization in $\tau_{fixed}$ and $\tau_{ready}$ where the task can be performed on the processor with the capacity of *p.c*. The function *getFirstLocalUtilizationTask*($\tau_{fixed},$ $\tau_{ready}$) returns the task with the highest local utilization in $\tau_{fixed}$ and $\tau_{ready}$. The function *allocateTaskToProcessor*(τ, *p*) assigns the task τ to the processor *p*.

**Algorithm 2** Classification of selected processors for scheduling  1:**Input**: $P_{selected},\tau_{ready}$  2:**Output**: $P_{fixed},P_{max},P_{slack},\tau_{fixed},\tau_{max},\tau_{slack}$  3:$P_{fixed}$—The set of processors fixed by a task  4:$P_{max}$—The set of processors having maximum utilization  5:$P_{slack}$—The set of processors to be able to have slack time  6:$\tau_{fixed}$—The set of tasks fixed to a processor on on $P_{fixed}$  7:$\tau_{max}$—The set of tasks scheduled on $P_{max}$  8:$\tau_{slack}$—The set of tasks scheduled on $P_{slack}$  9:$\tau_{1}$—Temporary variable for tasks10:$\tau_{2}$—Temporary variable for tasks11:$p_{1}$—Temporary variable for processors12:$p_{2}$—Temporary variable for processors13:**repeat**14:    $\tau_{1}$ = *getFirstLocalUtilizationTask*($\tau_{ready}$);15:    *availableCapacity* = $\sum_{p_{i}\in P_{slack}}p_{i}.c-\sum_{\tau_{i}\in \tau_{slack}}\tau_{i}.r\left(t_{0}\right)$;16:    $p_{1}$ = *getMinimumCapacityProcessor*($availableCapacity,\tau_{1},P_{selected}$);17:    **if** $p_{1}.c=\tau_{1}.r(t_{0})$ **then**18:        add($p_{1},P_{fixed}$);19:        add($\tau_{1},\tau_{fixed}$);20:    **else if** *availableCapacity* = 0 **then**21:        **for** $\forall \tau_{2}\in \tau_{slack}$ **do**22:           add($\tau_{2},\tau_{max}$);23:           erase($\tau_{2},\tau_{slack}$);24:        **end for**25:        **for** $\forall p_{2}\in P_{slack}$ **do**26:           add($p_{2},P_{max}$);27:           erase($p_{2},P_{slack}$);28:        **end for**29:        add($\tau_{1},\tau_{slack}$);30:        add($p_{1},P_{slack}$);31:    **else**32:        add($\tau_{1},\tau_{slack}$);33:        add($p_{1},P_{slack}$);34:    **end if**35:    erase($p_{1},P_{selected}$);36:**until**$\tau_{1}$ is not null37:**return** $P_{fixed},P_{max},P_{slack},\tau_{fixed},\tau_{max},\tau_{slack}$


Algorithm 4 shows the movement of the elements between processor sets and task sets. When an event-b occurs, all the tasks which have triggered an event-b are moved to $\tau_{done}$ and are removed from $\tau_{active}$. The function *getEventTasks*() returns all the tasks that have triggered the event-b. When an event-c or an event-f occurs, all the tasks that have triggered the event are moved to $\tau_{fixed}$, and the processors that have triggered the event are moved to $P_{fixed}$. The function *getProcessor*($\tau .r(t_{0})$, $P_{max}$) returns the processor with the capacity $\tau .r(t_{0})$ in $P_{max}$. When an event-t occurs, the processors which can be switched to sleep mode are moved to $P_{sleep}$ and are removed from $P_{slack}$. When an event-s or an event-r occurs, all the tasks that have triggered the event are moved to $\tau_{done}$ and are removed from $\tau_{active}$. The function *reallocateProcessorTime*() assigns the available processing time to a task with remaining execution time in $\tau_{done}$. The assigned task is moved to $\tau_{ready}$.

**Algorithm 3** Assignment of tasks to processors at rescheduling  1:**Input**: $P_{fixed},P_{max},P_{slack},\tau_{fixed},\tau_{max},\tau_{slack}$  2:**Output**: $\tau_{fixed},\tau_{max},\tau_{slack}$  3:**for** $\forall \tau \in \tau_{active}$ **do**  4:    *add*($\tau ,\tau_{ready}$);  5:**end for**  6:*eraseAll*($\tau_{active}$);  7:**for** $\forall p\in P_{slack}$ **do**  8:    τ = *getMaximumLocalUtilizationTask*($p.c,\tau_{slack},\tau_{ready}$);  9:    **if** τ is null **then**10:        τ = *getFirstLocalUtilizationTask*($\tau_{slack},\tau_{ready}$);11:    **end if**12:    *allocateTaskToProcessor*(τ,p);13:    *erase*($\tau ,\tau_{ready}$);14:    *add*($\tau ,\tau_{active}$);15:**end for**16:**for** $\forall p\in P_{max}$ **do**17:    τ = *getMaximumLocalUtilizationTask*($p.c,\tau_{max},\tau_{ready}$);18:    **if** τ is null **then**19:        τ = *getFirstLocalUtilizationTask*($\tau_{max},\tau_{ready}$);20:    **end if**21:    *allocateTaskToProcessor*(τ,p);22:    *erase*($\tau ,\tau_{ready}$);23:    *add*($\tau ,\tau_{active}$);24:**end for**25:**for** $\forall p\in P_{fixed}$ **do**26:    τ = *getMaximumLocalUtilizationTask*($p.c,\tau_{fixed},\tau_{ready}$);27:    *allocateTaskToProcessor*(τ,p);28:    *erase*($\tau ,\tau_{ready}$);29:    *add*($\tau ,\tau_{active}$);30:**end for**31:**return** $\tau_{fixed},\tau_{max},\tau_{slack}$


**Figure 4:**
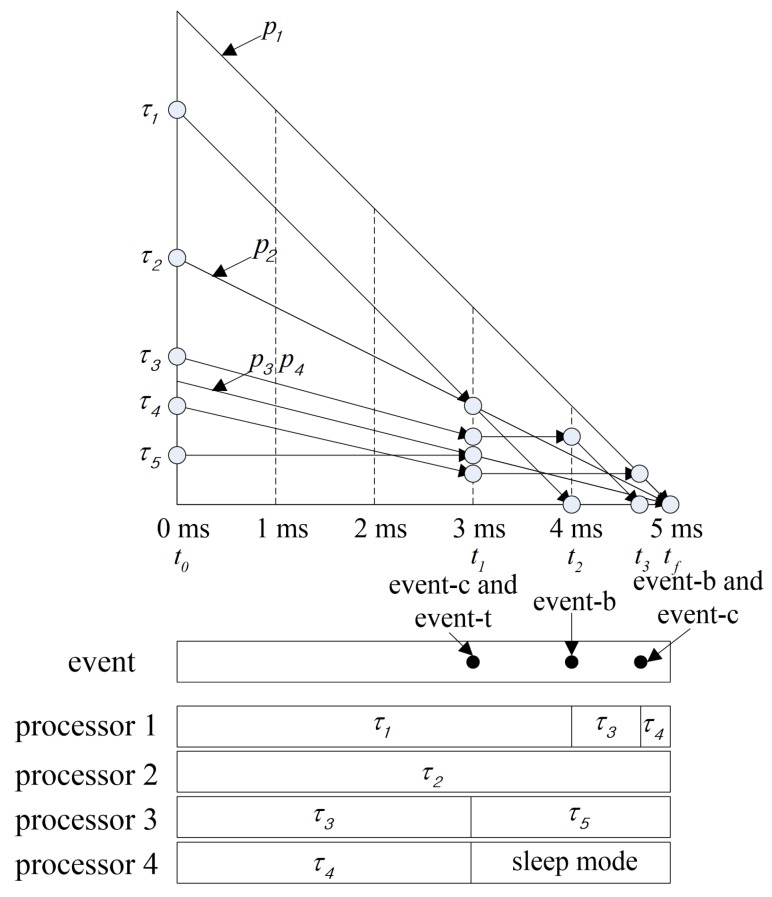
A scheduling in the first plane.

Figure 4 shows the scheduling in the first plane from the proposed method when scheduling the tasks of Table 8 on the processors listed in Table 9. Algorithm 2 is used to categorize the processor sets and ready tasks selected by Algorithm 1 at $t_{0}$. Task $\tau_{5}$ that has triggered an event-c at $\tau_{1}$ and the processor $p_{3}$ whose capacity is equal to the local utilization of $\tau_{5}$ are moved to $\tau_{fixed}$ and $P_{fixed}$, respectively. At the same time, the processor $p_{4}$ is moved to $P_{sleep}$ by event-t. Task $\tau_{1}$ that has triggered an event-b at $t_{2}$ is moved to $\tau_{done}$. Task $\tau_{3}$ that has triggered an event-b at $t_{3}$ is moved to $\tau_{done}$. At the same time, task $\tau_{3}$ that has triggered an event-c and the processor $p_{1}$ whose capacity is equal to the local utilization of $\tau_{3}$ are moved to $\tau_{fixed}$ and $P_{fixed}$, respectively. Table 10 shows the elements added to the processor and task sets by Algorithm 4 at each event in the *1st* plane. Tasks are assigned to processors by Algorithm 3. As shown in Figure 4, tasks assigned to processors move diagonally along the slope of the processor capacity and tasks unassigned to processors will move horizontally.

**Algorithm 4** Movement of elements during rescheduling in the T-L plane  1:**Input**: $P_{fixed},P_{max},P_{slack},\tau_{fixed},\tau_{max},\tau_{slack}$  2:**Output**: $P_{sleep},\tau_{ready},\tau_{active},\tau_{done}$  3:$\tau_{active}$—The set of tasks to be excuted  4:$\tau_{done}$—The set of tasks to be done  5:**if** event-b **then**  6:    *T* = *getEventbTasks*();  7:    **for** ∀τ∈T **do**  8:        *add*($\tau ,\tau_{done}$);  9:        *erase*($\tau ,\tau_{active}$);10:    **end for**11:**else if** event-c ∣ event-f **then**12:    *T* = *getEventcOrEventfTasks*();13:    **for** ∀τ∈T **do**14:        *add*($\tau ,\tau_{fixed}$);15:        **if** $\tau \in \tau_{max}$ **then**16:           *p* = *getProcessor*($\tau .r\left(t_{0}\right),P_{max}$);17:           *erase*($\tau ,\tau_{max}$);18:           *erase*($p,P_{max}$);19:        **else**20:           *p* = *getProcessor*($\tau .r\left(t_{0}\right),P_{slack}$);21:           *erase*($\tau ,\tau_{slack}$);22:           *erase*($p,P_{slack}$);23:        **end if**24:        *add*($p,P_{fixed}$);25:    **end for**26:**else if** event-t **then**27:    *capacity* = $\sum_{p_{i}\in P_{slack}}p_{i}.c-\sum_{\tau_{i}\in \tau_{slack}}\tau_{i}.r\left(t_{0}\right)$28:    *p* = *getProcessor*(*capacity*, $P_{slack}$);29:    *add*($p,P_{sleep}$);30:    *erase*($p,P_{slack}$);31:**else if** event-s ∣ event-r **then**32:    *T* = *getEventsOrEventrTasks*();33:    **for** ∀τ∈T **do**34:        *add*($\tau ,\tau_{done}$);35:        *erase*($\tau ,\tau_{active}$);36:    **end for**37:    *reallocationProcessorTime*();38:    **for** $\forall \tau \in \tau_{done}$ **do**39:        **if** $\tau .l(t_{cur})=0$ **then**40:           continue;41:        **else**42:           *add*($\tau ,\tau_{ready}$);43:           *erase*($\tau ,\tau_{done}$);44:        **end if**45:    **end for**46:**end if**47:**return** $P_{sleep},\tau_{ready},\tau_{active},\tau_{done}$


## 4. Energy Efficiency on Uniform Multi-Processors

**Figure 5:**
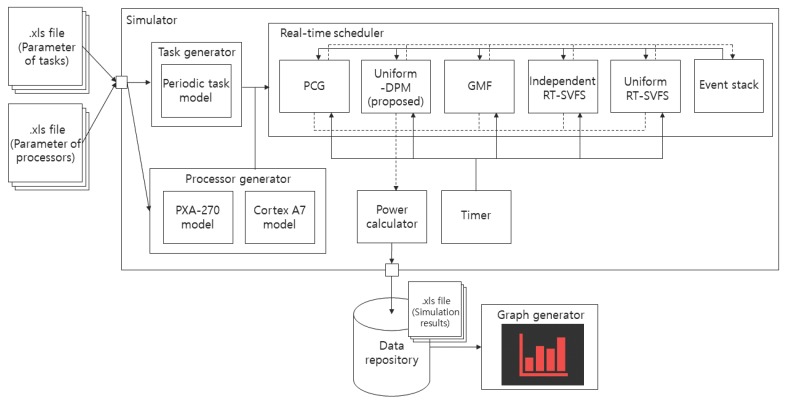
The architecture of the simulator.

In this section, the performance of the proposed algorithm is compared with the major algorithms previously developed for power management. We implemented a simulator operating in Windows 10 using the Ruby language (version 2.4.1) for the experiments. Figure 5 illustrates the architecture of the simulator. The results of the simulation show the energy consumption for task executions, as well as the energy overheads associated with the state transitions.

### 4.1. Experiment Environment

The characteristics of the cortex-A7 core in Marvell’s MV78230, which is the Multi-Core ARMv7 system based on the chip processor, is used to set the experimental parameters of the processor in the simulator. This core supports dynamic frequency scaling and dynamic power down options. Table 11 and Table 12 show that cortex-A7 supports six frequency levels and five processor states. Run thermal is used in the stress test of the CPU. The deep idle and sleep modes consume the same energy with respect to the CPU. We consider the run typical, idle, and sleep modes in Table 12 for our experiment. WolfBot [16], which is a distributed mobile sensing platform, has ARMv7 based cortex processors.

To confirm the scalability of the proposed algorithm, we change the number of available processors within the range 8–32. Then, we use the Emberson procedure to construct 100 task sets on each available processor. The total utilization of the task set is equal to 8, and the task has a utilization within 0.01–0.99. The period of each task is evenly distributed within 10–150 and simulated for 1000 system units.

### 4.2. Experiment Results and Analysis

Table 13 shows the platform type and power management technique of the algorithm to be simulated. The algorithm’s platform type is called “non-uniform” when the associated frequency of each processor is independently adjustable, and is called “uniform” when it can change all the frequencies at a constant rate when scaling the frequency of the processor. It is possible for each processor among the uniform multi-processors to operate at a different frequency. A job has a different execution time depending on which processor is allocated. These platforms are otherwise called “unrelated”.

**Figure 6:**
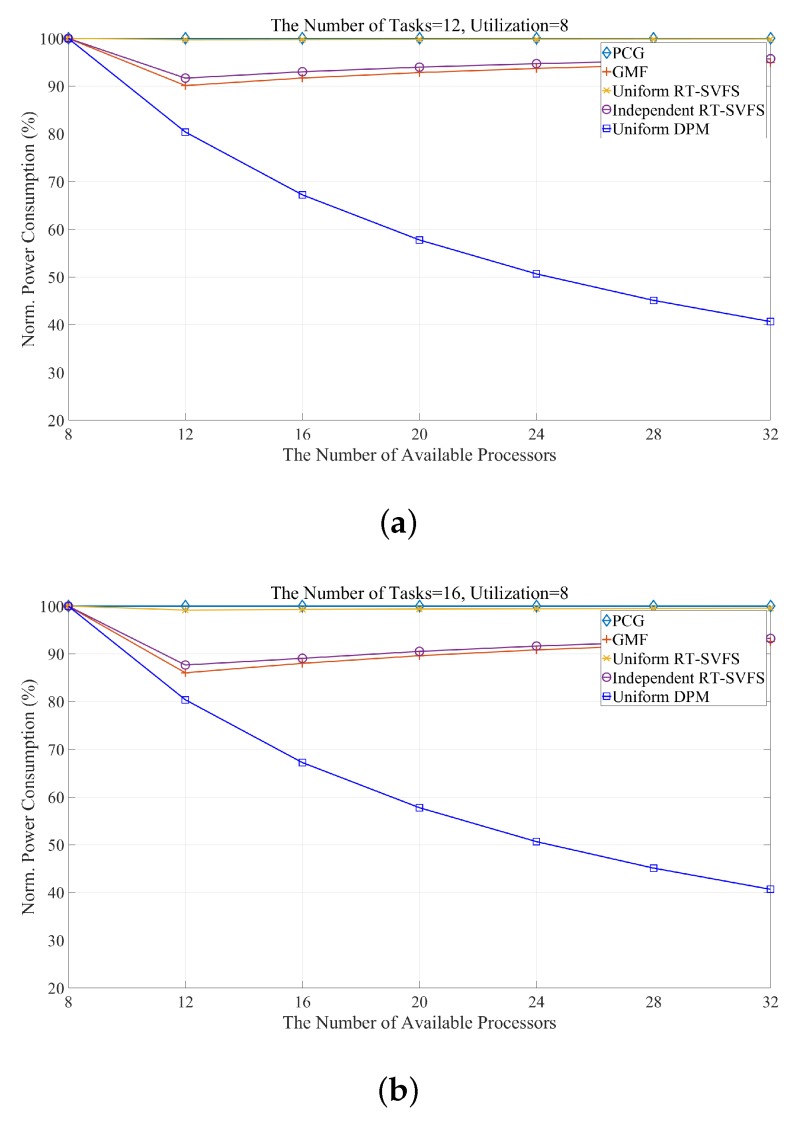

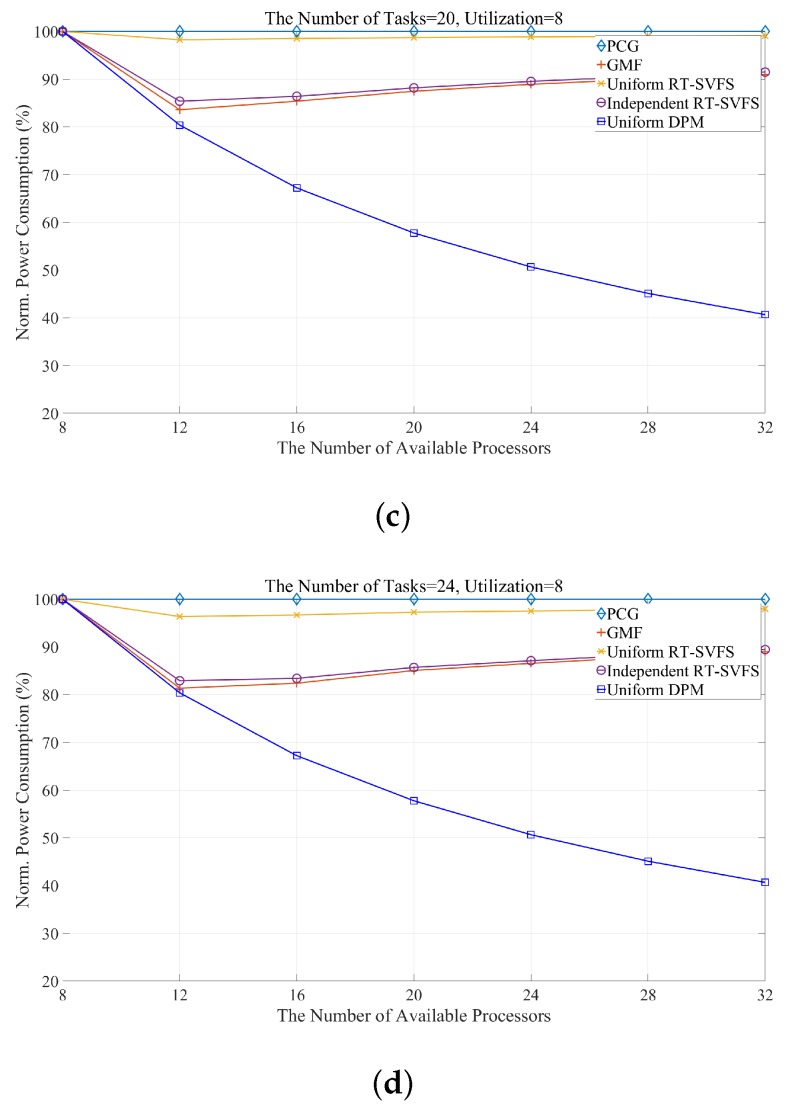
Comparing the energy consumption of an energy-efficient approach while varying the number of tasks: (**a**) 12; (**b**) 16; (**c**) 20; and (**d**) 24.

Figure 6 shows the power efficiency obtained by simulating the five algorithms mentioned in Table 13 while varying the number of available processors and the number of tasks. We implement our proposed algorithm as well as the following models: PCG, the original uniform algorithm without any power management [29]; Uniform-DPM, our proposed scheduling algorithm for DPM-embedded uniform multi-processors; GMF [31]; Independent RT-SVFS [30]; and Uniform RT-SVFS [30]. The *x*-axis of Figure 6 represents the number of available processors, and the *y*-axis represents the normalized power consumption (NPC). The power consumption consumed by the PCG is measured by the reference consumption and the power consumption rate of each algorithm. Figure 6 show the results when the number of tasks composing a task set is 12, 16, 20, and 24, respectively. All of the algorithms to be simulated is global optimal scheduling. Thus, since the total utilization of the task set used in the simulation is fixed at 8, the power efficiency of all algorithms shows 100% energy consumption in all scheduling using eight processors. As shown in Figure 6, the GMF and RT-SVFS algorithms change the power efficiency according to the number of tasks, while the proposed algorithm, Uniform-DPM, consumes the same a mount of power. This is because they always generate the same idle time. In addition, in the case of many available processors, the proposed algorithm shows high power efficiency by preventing unnecessary processor activation and idle time fragmentation, and by preventing frequent state transitions of the processor. GMF and independent RT-SVFS have similar power efficiencies because they determine the frequency of each processor independently. GMF finds a global optimal solution in the search spaces, but not Independent-SVFS. Thus, GMF is better than Independent-SVFS, as shown in Figure 6. Uniform RT-SVFS adjusts the frequency of all processors to a certain ratio, so if the number of tasks is small, the energy efficiency is not good because the work can be concentrated on some processor and the frequency of the processor cannot be lowered. However, as the number of tasks increases, the number of tasks can be divided and processed simultaneously by multiple processors, which can reduce the frequency of the processor. Table 14 and Table 15 show the energy efficiency characteristics of this proposed algorithm. Table 14 shows that Uniform-DPM always shows constant energy efficiency regardless of the number of tasks. Table 15 shows that the energy efficiency increases as the number of processors increases.

## 5. Conclusions and Future Works

The lifetime of WSNs is closely related to the management of sensor nodes operating at limited energy. In this paper, we propose a power management method for sensor nodes supporting DPM-enabled uniform multi-processors. In the proposed approach, the selection of processors to process a set of tasks and the assignment of tasks to the selected processors have been proposed in terms of energy efficiency. In addition, we implement a simulator to measure the power consumption of various scheduling algorithms. The experimental results show that the proposed algorithms provide better scalability to the number of available processors than DVFS-based approaches. Currently, our proposed algorithms can handle periodic tasks with implicit deadlines. In future work, we plan to extend our algorithms to handle sporadic tasks with time constraint. We are very interested in combining the DVFS and DPM approaches for T-L plane abstraction as well. In addition, studies on trade-offs between the power usage and computational complexity, as well as performance evaluations on overloaded situations, would be interesting problems for potential future research.

## Figures and Tables

**Table 1:** Mobile sensing platforms.

Platform Name	Processor Type	Sensor Type	Battery Type
R-One [11]	ARM Cortex-M3	Accelerometer, gyroscope, bump, IR, ambient light	3.7 V lithium-ploymer battery with 2000 mAh
E-puck [12]	dsPIC 30F6014A	IR, accelerometer, microphone	Battery swappable and rechargeable with 5 Wh
MarXBot [13]	ARM11	IR, camera, accelerometer, gyroscope, RFID, 2D force, microphone	Hot-swappable battery with 38 Wh
Foot-Bot [14]	i.MX31 ARM11	IR, camera	3.7 V lithium-polymer battery with 10-Ah
CITRIC [15]	Xscale PXA-270	Camera, microphone	Four AA batteries
WolfBot [16]	ARM Cortex-A8	IR, camera, microphone, ambient light, accelerometer, magnetometer	7.4 V lithium-ion battery with 5200 mAh

**Table 2:** An example of the available processor sets.

	$S_{1}$	$S_{2}$	$S_{3}$
$p_{1}\;$(voltage = 1.4 v, freq. = 600 MHz, capacity = 1)	O	X	O
…	X	X	X
$p_{5}\;$(voltage = 1.2 v, freq. = 300 MHz, capacity = 0.5)	X	O	O
$p_{6}\;$(voltage = 1.2 v, freq. = 300 MHz, capacity = 0.5)	X	O	X
…	X	X	X
$p_{n-1}\;$(voltage = 1 v, freq. = 150 MHz, capacity = 0.25)	O	O	X
$p_{n}\;$(voltage = 1 v, freq. = 150 MHz, capacity = 0.25)	O	O	X
…	X	X	X
total capapcity	1.5	1.5	1.5

**Table 3:** Task properties.

Task	Period	WCET	Utilization
$\tau_{1}$	5 ms	2.5 ms	0.5
$\tau_{2}$	10 ms	5 ms	0.5
$\tau_{3}$	10 ms	1.25 ms	0.25
$\tau_{4}$	20 ms	2.5 ms	0.25

**Table 4:** Dynamic power consumption of some feasible processor sets.

	$S_{1}$	$S_{2}$	$S_{3}$
Dynamic power consumption	2.46α*C*	1.94α*C*	2.68α*C*

**Table 5:** Processor properties.

	$p_{1}$	$p_{2}$	$p_{3}$	$p_{4}$
Supply voltage	1.4 V	1.2 V	1.0 V	1.0 V
Operating frequency	600 MHz	300 MHz	150 MHz	75 MHz
Processing capacity	1	0.5	0.25	0.125

**Table 6:** Selecting processors for scheduling a task set.

	$S_{1}$	$S_{2}$	$S_{3}$	$S_{4}$	$S_{5}$	$S_{6}$	$S_{7}$	$S_{8}$	…
$p_{1}$	O	O	O	O	X	O	O	X	…
$p_{2}$	O	O	O	X	O	O	X	O	…
$p_{3}$	O	O	X	O	O	X	O	O	…
$p_{4}$	O	X	O	O	O	X	X	X	…
Total capacity	1.875	1.75	1.625	1.375	0.875	1.5	1.25	0.75	…

**Table 7:** Task properties.

Task	Period	WCET	Utilization
$\tau_{1}$	5 ms	4.5 ms	0.9
$\tau_{2}$	10 ms	4.25 ms	0.425

**Table 8:** Task properties.

Task	Period	WCET	Utilization
$\tau_{1}$	5 ms	4 ms	0.8
$\tau_{2}$	5 ms	2.5 ms	0.5
$\tau_{3}$	10 ms	3 ms	0.3
$\tau_{4}$	10 ms	2 ms	0.2
$\tau_{5}$	20 ms	2 ms	0.1

**Table 9:** Processor properties.

	$p_{1}$	$p_{2}$	$p_{3}$	$p_{4}$
Supply voltage	1.4 V	1.2 V	1.0 V	1.0 V
Processing capacity	1	0.5	0.25	0.25

**Table 10:** Example of sets at events in the plane.

Set	Element			
	$t_{0}$	$t_{1}$	$t_{2}$	$t_{3}$
$\tau_{fixed}$	$\tau_{2}$	$\tau_{2}$, $\tau_{5}$	$\tau_{2}$, $\tau_{4}$	$\tau_{2}$, $\tau_{4}$, $\tau_{5}$
$P_{fixed}$	$p_{2}$	$p_{2}$, $p_{3}$	$p_{2}$, $p_{3}$	$p_{1}$, $p_{2}$, $p_{3}$
$\tau_{max}$	.	.	.	.
$P_{max}$	.	.	.	.
$\tau_{slack}$	$\tau_{1}$, $\tau_{3}$, $\tau_{4}$, $\tau_{5}$	$\tau_{1}$, $\tau_{3}$, $\tau_{4}$	$\tau_{3}$, $\tau_{4}$	.
$P_{slack}$	$p_{1}$, $p_{3}$, $p_{4}$	$p_{1}$	$p_{1}$	.
$\tau_{done}$	.	.	$\tau_{1}$	$\tau_{1}$, $\tau_{3}$
$P_{sleep}$	.	$p_{4}$	$p_{4}$	$p_{4}$

**Table 11:** Frequency levels of the cortex-A7 core.

Parameter	Level 1	Level 2	Level 3	Level 4
Frequency (MHz)	800	1066	1333	1600
Run typical power (W)	3.3	3.6	4	4.9

**Table 12:** Power states of the cortex-A7 core.

States	Power (Watts)
Run Thermal	5.9
Run Typical	4.9
Idle	2.4
Deep Idle	0.07
Sleep	0.07

**Table 13:** Summary of the energy-efficient scheduling algorithms.

Algorithm Name	Platform Type	Power Management
PCG	Uniform	-
Uniform-DPM (proposed)	Uniform	DPM
GMF	Non-uniform	SVFS
Independent RT-SVFS	Non-uniform	SVFS
Uniform RT-SVFS	Uniform	SVFS

**Table 14:** Summary of the experimental results by varying the number of tasks.

		Saved Norm. Power Consumption (%)			
**# of Processors**	**# of Tasks (Total Untilization)**	**Uniform-DPM**	**GMF**	**Independent RT-SVFS**	**Uniform RT-SVFS**
12	12 (8)	19.6	9.9	8.3	0.3
12	16 (8)	19.6	14	11	0.8
12	20 (8)	19.6	16.4	14.6	1.7
12	24 (8)	19.6	18.6	17	3.6

**Table 15:** Summary of the experimental results by varying the number of uniform processors.

		Saved Norm. Power Consumption (%)			
**# of Processors**	**# of Tasks (Total Untilization)**	**Uniform-DPM**	**GMF**	**Independent RT-SVFS**	**Uniform RT-SVFS**
12	24 (8)	19.6	18.4	17.1	3.6
16	24 (8)	32.8	17.6	16.4	3.3
20	24 (8)	42.2	15	14.3	2.7
24	24 (8)	49.4	14.5	12.9	2.5
28	24 (8)	54.9	12.1	11.6	2.2
32	24 (8)	59.3	11	10.6	2
